# Differential Gray Matter Vulnerability in the 1 Year Following a Clinically Isolated Syndrome

**DOI:** 10.3389/fneur.2018.00824

**Published:** 2018-10-11

**Authors:** Ismail Koubiyr, Mathilde Deloire, Pierrick Coupé, Cécile Dulau, Pierre Besson, Amandine Moroso, Vincent Planche, Thomas Tourdias, Bruno Brochet, Aurélie Ruet

**Affiliations:** ^1^Univ. Bordeaux, Bordeaux, France; ^2^Inserm U1215 - Neurocentre Magendie, Bordeaux, France; ^3^CHU de Bordeaux, Bordeaux, France; ^4^Laboratoire Bordelais de Recherche en Informatique, UMR CNRS 5800, PICTURA, Talence, France; ^5^AixMarseille Univ, CNRS, CRMBM UMR 7339, Marseille, France

**Keywords:** multiple sclerosis, MRI, diffusion tensor imaging, gray matter, atrophy, clinically isolated syndrome, longitudinal

## Abstract

**Background and purpose:** Whether some gray matter (GM) regions are differentially vulnerable at the early stages of MS is still unknown. The objective of this study is to investigate whether deep and cortical GM are differentially vulnerable after a clinically isolated syndrome (CIS) suggestive of multiple sclerosis (MS).

**Methods:** Fifty-six patients with CIS (PwCIS) and 38 healthy controls (HC) had conventional and diffusion tensor imaging (DTI) at baseline and 46 PwCIS and 20 HC were rescanned after 1 year. Deep GM (DGM) volumes, cortical thickness (CTh), and DTI metrics (FA: fractional anisotropy; MD: mean diffusivity) within these structures were calculated for each participant at each time-point and compared between PwCIS and HC. Linear regression models were used to investigate whether baseline DTI parameters could predict GM volume loss over time.

**Results:** At baseline, GM volumes did not differ between PwCIS and HC, but hippocampal MD was higher in PwCIS than HC (*p* < 0.01). Over 1 year, GM alterations became more widespread with putamen and hippocampus volumes decreasing in PwCIS (*p* < 0.01), and cortical thinning in different parts of the cortex along with a significant increase of MD. Hippocampus MD at baseline could predict its volume loss (*R*^2^ = 0.159; *p* < 0.05) and cortical thinning was associated to microstructural damage (Spearman's rho ranging from −0.424 to −0.603 with *p* < 0.003).

**Conclusion:** Along with MS being a diffuse inflammatory disease, GM showed a differential vulnerability at the early stage spreading from hippocampus to the cortex. Hippocampus volume loss could be predicted by its MD at baseline.

## Introduction

Multiple sclerosis (MS) is an inflammatory, demyelinating and neurodegenerative disease of the central nervous system, leading to physical deterioration and cognitive impairment. At the clinical onset of the disease, approximately 85% of patients experience a monophasic neurological episode, known as a clinically isolated syndrome (CIS) (1). Gray matter (GM) atrophy has been found to occur in different phenotypes of MS associating deep GM (DGM) atrophy and cortical atrophy (2). GM atrophy has been shown to progress in the first years after the CIS, (3, 4) but conflicting results have been reported at the initial time of CIS, which questions whether or not the disease induces tissue loss from this very early stage (2, 5–7). Especially, DGM atrophy has been inconsistently found in CIS (8–10), while cortical atrophy seems to be absent (10). The dynamics of GM vulnerability at the early stages remain unclear, and the mechanisms leading to atrophy are not well understood.

A relationship has been suggested between atrophy in some GM nuclei and lesions in the related white matter tracts through Wallerian degeneration as this has been shown for the thalamus (11). However, a direct injury of the GM by inflammation is also possible, (12) as suggested by recent studies using magnetic resonance-positron emission tomography (13). Either mechanism could lead to some differential vulnerability of the GM as some structures might be more connected than others, or some types of neurons might be more fragile than others. This selective vulnerability has been shown in Alzheimer's disease particularly, as the pathology seems to spread from entorhinal cortex to hippocampus (14).

In order to study the selective vulnerability of GM, diffusion tensor imaging (DTI) allows to explore the microstructural integrity of the structures. A few studies used this technique in CIS, showing abnormal results in the thalamus (15), hippocampus (16), and the cerebellum (17) suggesting microstructural changes from the early stages of MS. In the other hand, cortex has been investigated in patients with MS with fractional anisotropy (FA) and mean diffusivity (MD) (18–20). Results of these studies presented some discrepancies, as FA in the normal appearing gray matter (NAGM) was found to be increased (19) or decreased, (18, 20) while MD either increased (18, 20) or showed no difference compared to healthy volunteers (19). Also, the majority of these findings was cross-sectional and could not infer about the dynamics of the microstructural damage spreading. Thus, gray matter microstructural damage that may be leading to irreversible atrophy is not well assessed in MS yet, and this even more for CIS patients.

The objective of this 1-year longitudinal study is to investigate the differential vulnerability of GM (both DGM and cortical GM) at the very early stages of MS. Both microstructural and macrostructural damage were assessed and we have investigated whether microstructural damage is able to predict future GM volume loss in PwCIS.

## Materials and methods

### Standard protocol, approvals, registration, and patient consent

Each participant gave written informed consent. Patients were included in a prospective study analyzing cognition in PwCIS (SCI-COG, ClinicalTrials.gov Identifier: NCT01865357). This study was approved by the local ethics committee.

### Participants

- Patients

Fifty-Six PwCIS were recruited less than 6 months after a first neurological episode suggestive of MS as defined by Thompson et al. (21) including optic neuritis, partial myelitis, supratentorial syndrome, brainstem, or cerebellar syndrome. The presence of at least two asymptomatic cerebral lesions on fast fluid-attenuated inversion recovery (FLAIR) images was required to confirm central nervous system involvement. Data were collected from December 2012 through January 2017. Each participant underwent an MRI scan. Forty-Six PwCIS were rescanned 1 year after the first assessment. Others patients declined to be rescanned. Exclusion criteria were age under 18, history of other neurological or psychiatric disorders, MS attack or corticosteroid pulse therapy within 2 months preceding screening, severe cognitive deficits (Mini-Mental State Examination <27), and severe depressive symptoms (Beck Depression Inventory score (BDI) > 27). Clinical assessment and the Expanded Disability Status Scale (EDSS) scores were determined by expert neurologists.

- Healthy controls

Thirty-eight healthy controls (HC) matched for age, sex, and educational level were also included and underwent an MRI scan. Twenty of these individuals were rescanned after 1 year.

### MRI acquisition

MRI acquisition was performed on a 3T MRI system at our MS center (Achieva TX system, Philips Healthcare, Best, The Netherlands; Signa, GE Healthcare, Discovery MR 750w, Milwaukee, Wisconsin). Seventeen patients (out of 46, i.e., 37%) were not scanned on the same machine at baseline and after 1 year (Philips Achieva at baseline and GE Discovery after 1 year). The acquisition protocol was harmonized between magnets and consisted of a three-dimensional (3D) T1-weighted sequence using magnetization prepared rapid gradient echo (MP-RAGE) imaging (TR = 8.2 ms, TE = 3.5 ms, TI = 982 ms, α = 7°, FOV = 256 mm, voxel size = 1 mm^3^, 180 slices), a two-dimensional (2D) FLAIR sequence (TR = 11000 ms, TE = 140 ms, TI = 2800 ms, FOV = 230 mm, 45 axial slices, 3-mm thick) and a diffusion tensor echo-planar-imaging (EPI) pulse sequence (TR = 11676 ms, TE = 60 ms, FOV = 230 mm, an isotropic resolution of 1.6 × 1.6 × 1.6 mm^3^ and *b* = 1000 s/mm^2^) in 21 non-colinear directions and one *b* = 0 s/mm^2^.

### MRI analyses

Lesions were segmented by the lesion growth algorithm as implemented in the Lesion Segmentation Tool (LST) version 2.0.15 (http://www.applied-statistics.de/lst.html) in Statistical Parametric Mapping (SPM12). It segments T1 images into 3 main tissue classes (cerebrospinal fluid (CSF), GM and WM). This information is then combined with the coregistered FLAIR to calculate lesion belief maps. An initial binary lesion map is first obtained by thresholding these maps with a prechosen initial threshold (kappa = 0.3). This map is subsequently grown along voxels that appear hyperintense in the FLAIR image. This results in a lesion probability map that is thresholded to 50% to obtain a lesions binary map. Finally, these maps were manually corrected by two blinded experts. Using these maps, a lesion filling algorithm (22) was applied to the T1-weighted images to avoid lesions that affect brain tissue segmentations.

For volumetric analyses of whole brain, total WM, total GM, total CSF and DGM structures, T1-weighted images were processed using volBrain (http://volbrain.upv.es). The segmentation procedure is described in detail elsewhere (23). Briefly, after denoising, and inhomogeneity correction, images were affine-registered into the Montreal Neurological Institute (MNI) space using Advanced Neuroimaging Tools (ANTs) (24), and the total brain volume was estimated. The hippocampus, caudate, putamen, thalamus, amygdala, accumbens, and globus pallidus were automatically segmented with a patch-based multi-templates method described in detail elsewhere (25) that uses expert manual segmentations in MNI space as priors. Every mask was then blindly checked and manually corrected if needed. To control for variations in head size, each structure's volume was assessed as a fraction of total intracranial volume (TIV). Cortical thickness (CTh) evaluation was performed with the Freesurfer 5.3 (26, 27) image analysis suite, which is documented and freely available online (http://surfer.nmr.mgh.harvard.edu). We performed the longitudinal stream (28) using an unbiased within-subject template space and image obtained by robust, inverse consistent registration (29). To detect possible misclassification of white and gray matter, all images were visually inspected. Cortical ribbon masks of the different lobes (frontal, temporal, parietal, occipital, cingulate, insula) were also extracted. Diffusion data were processed using the Oxford Centre for Functional MRI of the Brain (FMRIB) Software Library (FSL, version 5.0.9, fsl.fmrib.ox.ac.uk/fsl). Eddy current distortions and motion artifacts were first corrected (using the eddy_correct function), and the diffusion tensor was calculated. Scalar maps of fractional anisotropy (FA) and mean diffusivity (MD) were then extracted. Since no preferential water molecular motion is expected to occur in the GM, fractional anisotropy was used as a secondary metrics that is expected to be less sensitive.

To bring the GM masks and the scalar maps to the same space, we registered the T1-weighted images to the B0 image as a reference. This was done to keep the scalar maps in their native space, where the tensor was computed. To do that, we first used the FSL Brain Extraction Tool BET (30) (fsl.fmrib.ox.ac.uk/fsl/fslwiki/BET) to extract the brain from both the T1-weighted and the B0 images. Then, we resampled our B0 to 1 × 1 × 1 mm^3^. After that, we used a rigid registration followed by a non-rigid registration of the T1-weighted image to the subject's B0 space using ANTs (24). We then applied the transform obtained to our previous masks of GM (subcortical and cortical) and extracted DTI scalar parameters for each label. To avoid partial volume effect and the inclusion of cortical lesions, this cortical segmentation was masked by both the gray matter mask from volBrain and the segmented cortical lesions.

### Statistical analysis

Statistical analysis was performed using SPSS software version 23.0 (IBM SPSS Statistics for Macintosh, Version 23.0. Armonk, NY: IBM Corp).

All MRI measures analyses were statistically adjusted for the scanner. Normality of the distribution was assessed using the Shapiro-Wilk test. Parametric and non-parametric tests were used according to the distribution of the variables. Categorical variables were investigated with χ^2^ tests. At baseline, volumes, CTh, and DTI scalars comparisons between PwCIS and HC were performed by general linear models (GLM) where gender, age and level of education were entered as covariates. For the longitudinal comparisons of baseline and 1-year characteristics of our subjects, paired *t*-tests or Wilcoxon tests were used as appropriate. Relationships between imaging variables were assessed using correlation coefficients (Pearson or Spearman according to statistical distribution). To investigate whether baseline DTI parameters could predict volume loss during 1 year in DGM, DGM volume loss (dependent variable) was predicted with hierarchical regression models, including two hierarchical blocks. In the first block, age, gender, level of education and scanner also known as nuisance variables were systematically forced into the model. In the second block, the abnormal DTI metrics were added to the variables of the first block. Considering the issue of multiple comparisons, all the following results are Bonferroni corrected to reduce the risk of type I errors. A *p* < 0.05 was considered significant.

## Results

### Demographic and clinical characteristics

Clinical phenotypes of PwCIS were summarized in Table 1. PwCIS and HC groups were matched for age, gender and educational level at both time points. EDSS was not significantly different in the CIS group between the two time-points (Table 1).

**Table 1:** Demographic, clinical, and conventional MR imaging characteristics.

	**Baseline**		**Year 1**	
Clinical features	HC (*n* = 38)	CIS (*n* = 56)	HC (*n* = 20)	CIS (*n* = 46)
Mean age, years (SD)^a^	38.1 (9.3)	36.5 (11.2)	37.9 (8.4)	38.1 (11.5)
Sex ratio (F/M)^b^	26/12	46/10	14/6	36/10
Education level (high/low^c^)^b^	27/11	39/17	11/9	30/16
Symptoms at clinical onset: Brain Optic neuritis Brainstem/Cerebellar Spinal cord		5 (9%) 12 (21%) 11 (20%) 28 (50%)	–	–
Median EDSS score [range]^d^	-	1.0 [0–4]	-	1.0 [0–5]
Median T2-Lesion volume (ml)^d^	-	0.73 [0.23–63.12]	-	1.09 [0.61–67.74]
Normalized Brain fraction (%)^d,e,f^	86.36 ± 3.12	85.06 ± 3.93	86.39 ± 2.94	83.91 ± 4.03^*^^†^
Normalized WM fraction (%)^g,e,f^	37.02 ± 2.48	35.67 ± 3.17	37.32 ± 3.06	34.65 ± 3.33^†^
Normalized GM fraction (%)^g,e,f^	49.34 ± 2.64	49.40 ± 2.70	49.07 ± 2.26	49.26 ± 2.91
Normalized CSF fraction (%)^d,e,f^	13.64 ± 3.12	14.94 ± 3.93	13.61 ± 2.94	16.09 ± 4.03^*^^†^
Mean CTh (mm)^d,e^	2.55 ± 0.10	2.54 ± 0.12	2.54 ± 0.10	2.49 ± 0.12^†††^

a *Mann-Whitney test*.

b *χ^2^ test*.

c *Education level was considered as high or low according to a French baccalaureate*.

d *Wilcoxon test to compare PwCIS at baseline and year 1*.

e *GLM comparing PwCIS to HC with age, sex and level of education as covariates*.

f Percentage: (structure's volume/TIV)*100.

g *Paired t-test*.

*Differences between PwCIS and HC*,

* *p < 0.05*.

*Differences in PwCIS between baseline and year 1*,

† *p < 0.05*;

††† *p < 0.001*.

*TIV, Total Intracranial Volume; EDSS, Expanded Disability Status Scale; WM, White Matter; GM, Gray Matter; CSF, Cerebrospinal fluid (includes liquid in subarachnoid space and ventricular system); CTh, Cortical Thickness*.

In this cohort of patients, after 1 year, 65.2% of PwCIS were diagnosed with MS according to the 2010 McDonald criteria.

### Conventional MRI

T2-Lesion load (T2-LL) did not differ in the CIS group between the two time-points (Table 1).

At baseline, there was no difference between the two groups in whole normalized brain volume, WM, GM and total CSF. However, over 1 year, PwCIS showed a global brain atrophy as the whole brain volume and WM decreased (*p* < 0.05), (Table 1) and total CSF volume increased (*p* < 0.05).

### Baseline findings

We wanted to assess whether some GM regions are more vulnerable at the onset of the disease than others.

- Volumetric measures

We found no significant atrophy in DGM structures and no cortical thinning when comparing PwCIS to HC.

- DTI

**Figure 1:**
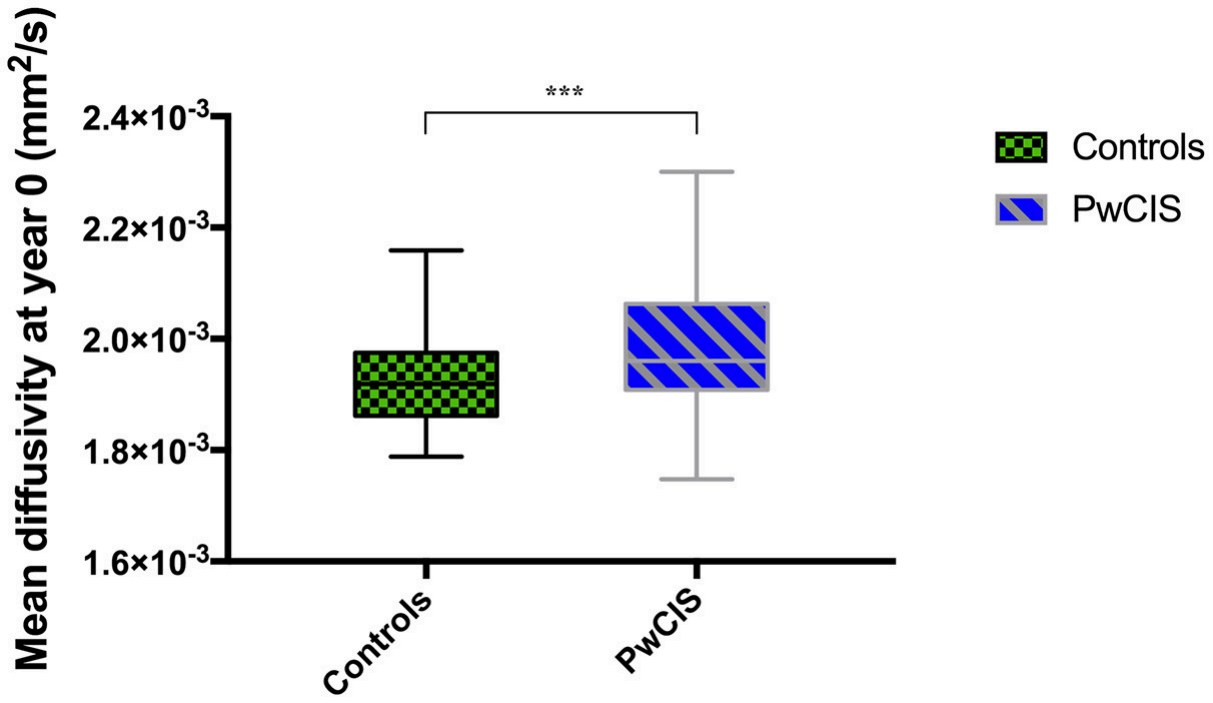
Hippocampus mean diffusivity at baseline. PwCIS, patients with clinically isolated syndrome. *p*-values indicate significant differences after multiple comparisons correction. ***p < 0.001.

However, when looking at the microstructural integrity of the GM, hippocampus was the only structure altered at this stage as its MD was significantly higher in PwCIS compared to HC (Figure 1), indicating a differential vulnerability of this structure compared to the rest of the GM.

### Longitudinal findings

To study the evolution of GM damage, we compared these structures between year 0 and year 1.

- Volumetric measures

**Figure 2:**
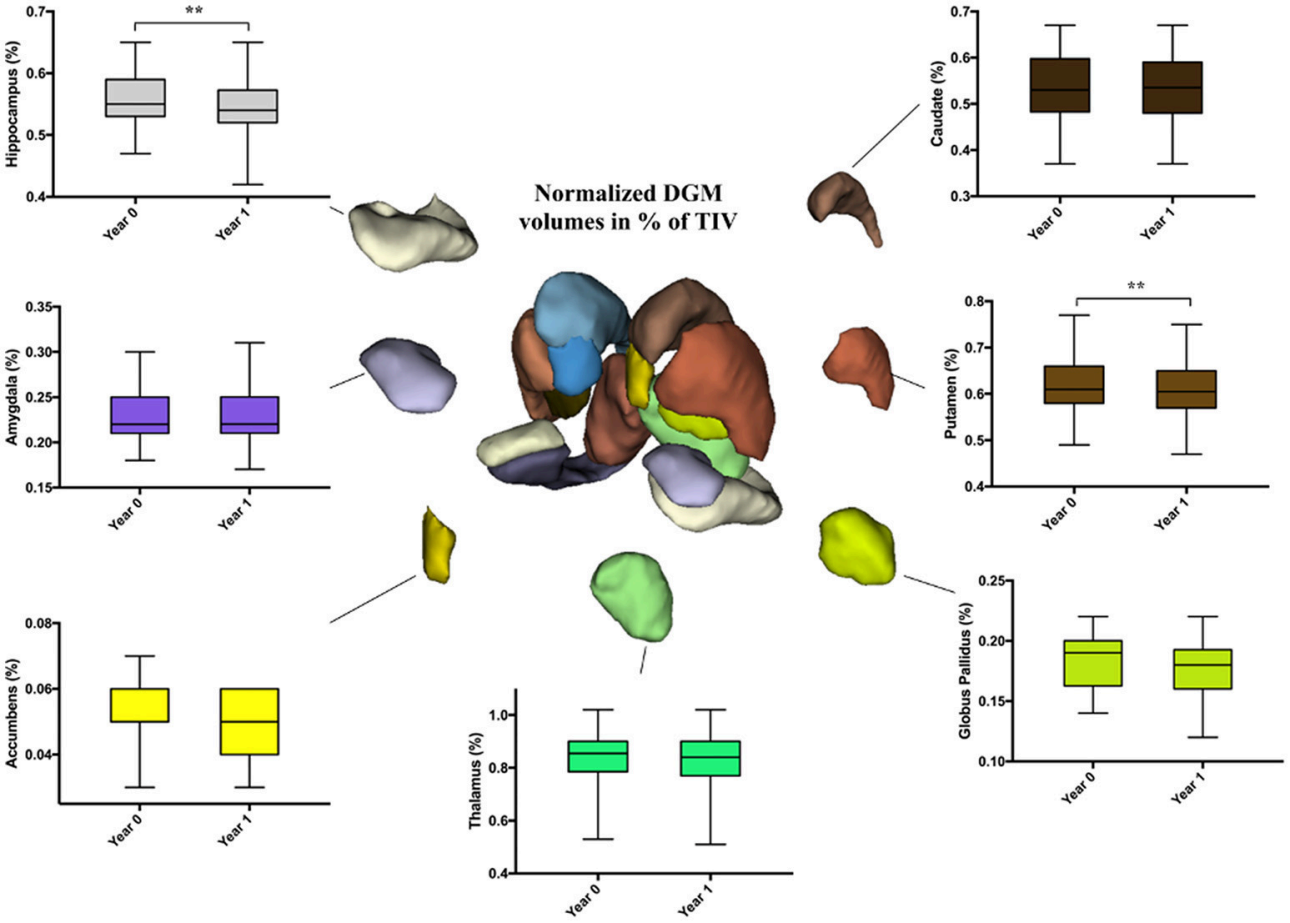
Longitudinal comparison of DGM structures volume fractions in PwCIS. Volumes are percentages calculated as: (structure's volume/TIV)*100. DGM, Subcortical deep gray matter; PwCIS, patients with clinically isolated syndrome. *p*-values indicate significant differences after multiple comparisons correction. **p < 0.01.

No differences were noticed in HC. However, in PwCIS, lateral ventricles volumes increased (*p* < 0.01) reflecting deep brain atrophy, whereas putamen and hippocampus volumes significantly decreased (Figure 2).

The mean CTh significantly decreased in 1 year (*p* < 0.001). Regionally, multiple brain areas of significant cortical thinning in both hemispheres were observed. This cortical thinning was noticed in the bilateral frontal and temporal lobes, left insula, and right parietal lobe (Table 2).

- DTI

**Table 2:** Cortical thinning in a longitudinal comparison of PwCIS between baseline and 1 year of follow-up.

**Regions**	**Baseline thickness (mm)**	**1-year thickness (mm)**
Frontal_L	2.52 ± 0.14	2.45 ± 0.14^***^
Cingulate_L	2.62 ± 0.23	2.66 ± 0.19
Occipital_L	2.01 ± 0.14	1.97 ± 0.12
Temporal_L	2.74 ± 0.20	2.67 ± 0.14
Parietal_L	2.31 ± 0.18	2.23 ± 0.13^**^
Insula_L	3.05 ± 0.18	2.99 ± 0.19^**^
Frontal_R	2.48 ± 0.15	2.37 ± 0.14^****^
Cingulate_R	2.57 ± 0.21	2.55 ± 0.17
Occipital_R	2.03 ± 0.17	1.99 ± 0.13
Temporal_R	2.79 ± 0.19	2.75 ± 0.13^**^
Parietal_R	2.31 ± 0.15	2.25 ± 0.12^**^
Insula_R	3.02 ± 0.18	2.94 ± 0.19

*L, Left hemisphere; R, Right hemisphere. p-values indicate significant differences after multiple comparisons correction*.

** *p < 0.01*;

*** *p < 0.001*;

**** *p < 0.0001*.

Regarding microstructural changes, PwCIS showed significantly higher MD in the left frontal (*p* < 0.006), left temporal (*p* < 0.003), left cingulate (*p* < 0.003) and bilateral parietal lobes (*p* < 0.002) (Table 3). No changes were noticed for the FA.

**Table 3:** Cortical mean diffusivity changes over 1 year of follow-up in PwCIS.

**Regions**	**Baseline MD (10^−3^ mm^2^/s)**	**1-year MD (10^−3^ mm^2^/s)**
Frontal_L	1.05 ± 0.13	1.11 ± 0.16^**^
Cingulate_L	0.93 ± 0.10	0.98 ± 0.11^**^
Occipital_L	0.89 ± 0.06	0.90 ± 0.07
Temporal_L	0.90 ± 0.05	0.92 ± 0.06^**^
Parietal_L	0.95 ± 0.11	1.01 ± 0.12^***^
Insula_L	1.03 ± 0.08	1.04 ± 0.10
Frontal_R	1.05 ± 0.12	1.11 ± 0.14
Cingulate_R	0.96 ± 0.09	0.99 ± 0.11
Occipital_R	0.90 ± 0.07	0.92 ± 0.07
Temporal_R	0.91 ± 0.06	0.92 ± 0.06
Parietal_R	0.96 ± 0.11	1.02 ± 0.13^**^
Insula_R	1.02 ± 0.08	1.03 ± 0.10

*MD, Mean diffusivity. L, Left hemisphere; R, Right hemisphere. p-values indicate significant differences after multiple comparisons correction*.

** *p < 0.01*;

*** *p < 0.001*.

### Relationship between volume loss and microstructural damage

While trying to assess whether microstructural damage at baseline could predict volume loss, we found that baseline hippocampus MD was able to predict hippocampus volume loss after 1 year (adjusted *R*^2^ = 0.16, *p* < 0.05) (Table 4). T2-LL was taken into account in this model and was not responsible for the volume loss.

**Table 4:** Hierarchical regression model between hippocampus volume loss over 1 year and demographical and baseline MD.

**Dependent variable**		**Explanatory variables**	**Univariate analysis (*r*)**	**Multivariate analysis (ß)**	**Adjusted multivariate model (*R*^2^)**
Hippocampus volume loss over 1 year	Block 1	Age	0.12	ns	ns
		Educational level	0.19	ns	
		Gender	0.05	ns	
		Scanner	0.05	ns	
	Block 2	Age	0.12	ns	0.16^*^
		Educational level	0.19	ns	
		Gender	0.05	ns	
		Scanner	0.05	ns	
		Hippocampus MD at year 0	−0.40^**^	−0.45^**^	

*MD, Mean diffusivity; ns, non-significant*.

* *p < 0.05*;

** *p < 0.01*.

We then investigated whether the microstructural abnormalities appearing over 1 year in the cortical ribbon could be related to the cortical thinning over the same period of time. Therefore, we found that the mean diffusivity change from year 0 to year 1 was strongly correlated to the cortical thinning in the left frontal lobe (rho = −0.603; *p* < 0.0001), the right parietal lobe (rho = −0.424; *p* < 0.003) and the left temporal lobe (rho = −0.427; *p* < 0.003).

We also investigated the relationship between EDSS and MRI (volumetric and DTI) abnormalities, but no correlation was found. These abnormalities were not able to predict the conversion to MS after 1-year.

## Discussion

The present study showed differential GM vulnerability as microstructural damage spread from the deep gray matter (hippocampus) to the cortex 1 year after the CIS. This microstructural damage occurred before irreversible gray matter atrophy. At baseline, there was no sign of GM atrophy in PwCIS compared to HC. However, microstructural damage was already present and the hippocampus was the first and only GM structure altered. The hippocampus showed increased MD in PwCIS compared to controls, confirming preliminary findings in a previous paper (16) that compared cross-sectionally a subgroup of this population with a group of MS patients. The damage occurring to the hippocampus was further confirmed by a significant volume loss after 1 year of follow-up. The putamen showed also a significant volume loss at follow-up. Since microstructural damage appears to precede volume loss, we investigated whether the former could predict the latter. We then showed that hippocampus MD was able to predict its volume loss independently of lesion volume. Microstructural abnormalities, as detected by DTI in the hippocampus, preceding volume loss and predicting it suggested that a primary tissue alteration within this structure is involved in the neurodegenerative process. Activation of microglia/macrophages, associated with demyelination and neuro degeneration, has been pathologically observed in DGM, including the hippocampus in MS (31–33). In experimental auto-immune encephalomyelitis, microglial activation within the hippocampus has been observed in association with neuronal dysfunction and memory impairment independently of demyelination (34). DTI was able to detect microstructural abnormalities in the hippocampus in this model. According to the pathological and experimental data discussed above, we hypothesized that neuro-inflammation that results in microglial activation, for example, could occur at the very early stages of the disease in the hippocampus. However, a role for lesions within the tracts connected to the hippocampus cannot be ruled out, but the persistence of the prediction after adjustment on lesion load suggests an independent mechanism.

Brain atrophy measured on MRI is considered as a hallmark of long lasting MS. It reflects the net effect of the pathology on the brain; it is correlated with physical and cognitive disability and increasingly used as an end-point in clinical trials. It is well-established that GM atrophy, including cortical and DGM atrophy, is mainly responsible for the development of the whole brain atrophy (35). The dynamics of GM atrophy remain, however, not well known and the mechanisms of GM alterations remain hypothetical. One possible mechanism is a consequence of distant lesions by dying back axonopathy, leading to atrophy of the cortical gray matter. However, it has also been suggested that meningeal inflammation and microglial activation could lead to direct pathology of the GM (36, 37) and some evidence of this mechanism has been observed in progressive MS (38).

We hypothesized that early structural abnormalities could be detected in some nuclei of DGM in CIS and could predict the subsequent development of atrophy in the same structures.

The different results between studies concerning the presence of DGM volume loss in CIS could be explained by different sample size, disease duration, and selection bias toward CIS with higher or lower burden of the disease. The disease duration in our sample was less than 6 months, and PwCIS must have only at least two lesions on the brain MRI; in fact, their lesion load was very low (0.7 cm^3^). For example, in one study showing some level of DGM atrophy (8), lesion volume was superior to 2 cm^3^. In another study, CIS patients with lesion load superior to 4.49 cm^3^ had lower DGM volumes than those with a median lesion load inferior to 4.49 cm^3^ (10). This suggests that in the CIS samples with lower lesion load, the pre-clinical stage before CIS was shorter, explaining why atrophy was not developed.

In our sample, we did not observe significant cortical thinning at baseline, in agreement with the absence of cortical atrophy in previous studies, (9) but we observed some cortical thinning during the follow-up period in bilateral frontal lobes, bilateral temporal lobes and left insular and right parietal lobes. These results are in line with the study by Rocca et al. (4) showing GM atrophy of frontal, temporal, and parietal lobes in CIS patients 1 year after the onset of the disease. Microstructural abnormalities in the cortex appeared only after 1 year of follow-up as opposed to hippocampus. MD of bilateral parietal lobes, left frontal, left temporal, and left cingulate lobes was significantly increased after 1 year in PwCIS. This result did not only reflect atrophy because of CSF contamination. First, we found an altered MD in hippocampus before any atrophy. Second, if an increase of MD in cortical GM was due to partial volume, we would also find a decrease of FA which was not the case. Moreover, these microstructural abnormalities were found to correlate with the presence of cortical thinning in left frontal and temporal lobes, as well as in the right parietal lobe. A recent study using 7 Tesla MRI showed the existence of a gradient of pathology in the cortex of MS patients, suggesting that the pathological process was driven from the pial surface (39) and supported the role of inflammation within the cortex in relation with meningeal inflammation. Cortical volume loss seems to parallel DGM volume loss at these very early stages of MS, however, microstructural damage starts within the hippocampus first.

The present study is not without limitations. First, our patients did not have double inversion recovery sequences, thus some cortical lesions may have been missed in the FLAIR sequence and included in the cortical gray matter mask. Second, we are aware that the follow-up time (1 year) is too short to observe more damage in our patients, thus we will follow them at a longer period of time. However, this short follow-up period was used to detect very early changes occurring at the onset of MS.

## Conclusion

The current study allowed us to explore the whole GM integrity and to detect differential vulnerability of the hippocampus at the earliest stage of MS, showing a pathological spread toward the cortex after 1 year of the disease.

Since the respective role of atrophy of cortical and DGM in clinical, physical and cognitive disability remains an important question, this cohort of CIS patients will be followed-up to explore this question.

## Ethics statement

This study was carried out in accordance with the recommendations of CPP Bordeaux Aquitaine with written informed consent from all subjects. All subjects gave written informed consent in accordance with the Declaration of Helsinki. The protocol was approved by the CPP Bordeaux Aquitaine.

## Data availability

The datasets generated during and/or analyzed during the current study are available from the corresponding author on reasonable request.

## Author contributions

IK and BB were involved in drafting the manuscript. All authors revised the manuscript for important intellectual content. MD, CD, AM, VP, TT, AR, and BB were involved in study concept and design. IK, MD, PC, CD, PB, AR, and BB were involved in analysis and interpretation of the data. IK, MD, CD, AM, VP, TT, AR, and BB were involved in acquisition of the data. IK and MD were involved in statistical analysis. BB, AR, and TT were involved in study supervision and coordination. All authors approved the final version to be published and agreed to be accountable for all aspects of the work in ensuring that questions related to the accuracy or integrity of any part of the work are appropriately investigated and resolved.

### Conflict of interest statement

CD reports personal fees from Biogen, outside the submitted work. VP reports non-financial support from Teva, non-financial support from Biogen Idec, personal fees and non-financial support from Merck-Serono, outside the submitted work. AR reports grants from TEVA, during the conduct of the study; personal fees and non-financial support from Novartis, personal fees and non-financial support from Biogen, grants, personal fees and non-financial support from TEVA, grants and non-financial support from Roche, grants, and non-financial support from Merck, grants and non-financial support from Genzyme, non-financial support from Medday, grants from Bayer, outside the submitted work. BB reports grants from French Ministry of Health, during the conduct of the study; personal fees and non-financial support from biogen-idec, grants from merck-serono, personal fees and non-financial support from novartis, personal fees, and non-financial support from genzyme, grants, personal fees, and non-financial support from teva, grants and non-financial support from bayer, outside the submitted work. IK, MD, PC, PB, AM, and TT have nothing to disclose.

## Abbreviations

CIS: clinically isolated syndrome
DGM: deep gray matter
PwCIS: patients with CIS
HC: healthy controls
FA: fractional anisotropy
MD: mean diffusivity
CTh: cortical thickness.
